# The Dentists of the Day

**Published:** 1889-05

**Authors:** J. B. Patrick


ARTICLE II.

### THE DENTISTS OF THE DAY.

### A STRIKING ILLUSTRATION OF THE PROGRESS OF THE AGE.

[Dr. J. B. Patrick's Address to the Graduating Class of the University of Maryland Dental Department.]

At the recent commencement of the University of Maryland, Dr. J. B. Patrick, of Charleston, S. C., delivered an address before the graduating class in dental surgery. The address was so admirable, both by reason of its interesting theme and by the manner of its delivery, that it attracted much favorable comment.

The address is as follows:

*Gentlemen*: To one who has crossed the equatorial line of life there is much significance in the opportunity that brings him face to face with the younger generation who are moving forward to complete what has possibly been left unfinished.

More than at any time in its history the world is now replete with growth. Science, art and literature have become factors in a progress for which history has no parallel. We are scanning nature through new lenses and enlarging old ideas with new mechanisms. In the field of electricity alone we have had occasion to pause and wonder, and the great heart of the world is still bounding with expectancy of the achievements yet to be.

From somewhere has come a marvellous impulse that is effecting a change in our habits, thoughts and methods of life. Men and women have higher aspirations than ever before. New policies are being presented, novel plans engage our attention, a process of intellectual crystallization is going on, and to the young people of to-day there opens a future grandly inviting and full of rewards, which to us of the past were unknown.

Do you wonder, then, that I feel a special pleasure in meeting you, gentlemen, on the threshold and in the vestibule of this hall? You are passing from an old and honored institution into a life-work. Before you are the examples of a splendid host, all of whom have made the names of your profession and this State illustrious. It is now your duty to show to the world that you inherit their worth, and are carrying forward the banner on which is inscribed their superb endeavor.

In the pursuit of your noble calling it is needless for me to say that the University of Maryland occupies a place abreast of the grandest institutions of its kind in the country. At home and abroad its name is a synonym for thoroughness in scholastic and practical training. Its graduates occupy high places. Their additions to medical, surgical and dental science are recorded in the official journals of America and Europe, and their achievements are entrenched among the memories of a people not slow to do honor. Around the names of Harris, Hayden, Austin, of the dental; Smith, Chew and McSherry, of the medical department, and a score of others, are clustered associations that will always remain tender, for these men were an "ever present help in time of need." They did not live in vain, and in our hearts their tombstones are always wreathed with flowers.

It is one of the wise sayings of India that even he who plants a tree has not lived in vain. The tree will yield fruit, or at least afford shade to those who will be born to-morrow, famished and naked. He who has planted a tree has done well; and he who cuts it down and has sawed it into planks has done well. He who puts the planks together to make a bench has done well; but he who, sitting upon the bench, has taken a child upon his knees and taught it to read, has done better than all the others. The first three have added something to the common capital of humanity. The last has added something to humanity itself. He has made man more enlightened.

In leaving your instructors in this institution, therefore, you have reason to recognize the fact that they have increased your power among the world's producers. They have added something to humanity. What they have reaped in other fields they have planted in you afresh in order that you in turn may go on sowing and reaping and grafting where you can the fruit of a useful life. Always, however, strive to maintain the integrity of the parent tree.

The history of civilization may be summarized in nine words: "The more one knows, the more one can do." It is the studious men of Christendom who, by a series of discoveries and inventions, have abridged and facilitated labor. The work done has helped the work to be done. It has been aptly said that the "tools of the human race are nothing else than a collection of ideas. All mechanisms are worn out in the long run. Steam engines are not everlasting and telegraphs may be taken down, but science, which is the soul of all these useful machines, will survive their destruction, and the idea that remains will enable us indefinitely to replace the material which perishes."

The first of useful things is man himself, and it is in the development of his facilities that will be found the power that makes the world move.

The professions that are organized in a struggle against the destructive forces which threaten us from birth afford a notable illustration of this thought. It is not less true of the department of dentistry established by the University of Maryland than of every other field of scientific labor. In fact you represent a profession in which the practical usefulness of its members is so frequently reflected upon the lives of others that its impairment or loss would be one of the most deplorable misfortunes to humanity. You are the embodiment, not of an ideal, but of substantial work, full of responsibilities and possibilities, which, according to the use you make of them, may influence your destiny and even the age in which you live. There is no intelligent life that is not contributive. There is no genuine effort made by a man who goes forth from an institution of this character with the superb equipment which it affords that is not manifold. He in person may not see the direct result, but if his duty be well and faithfully performed the influence he has generated will continue to radiate and leave their impress.

Your labor of life has therefore but just begun. Here only the substructures has been laid. The real edifice must be the work of your own hands, and in receiving these diplomas, which I know are conferred proudly and with many unuttered congratulations, remember that they are far more significant than any intrinsic value that may attach to them, for they tell the story of the high honor you have attained. They represent the blessing your alma-mater bestows, and, rightly respected, that blessing will always be a credential. You go forth from this institution with the stamp of its high approval; with the Godspeed of your instructors and the observant eyes upon you of your associates. In a large measure, you have been equipped from its armory for the battle of life, and if, when the guns are silenced and the smoke has cleared away, you have not added something, even though it be a trifle here and there, to the triumphs of human knowledge and achievement, you will have broken the record under which we so proudly write: "Well done, good and faithful servant."

The dentist of the present is unlike his less-experienced predecessor. His field is broader, his aims higher, his ambition is rewarded with grander results. He lives more in an atmosphere of science, and in nearer relationship to his patients. He is their confidant and friend, and the instances are numerous in which the greatest and best men and women of the land have sought to do him honor.

Voltaire describes the physician as "the man who pours drugs of which he knows nothing into bodies of which he knows less." Had he been able to penetrate the future he would recognize members of our profession as occupying a higher plane. The notion that the god of physic and the sender of diseases were both originally in the same trade has been dispelled, and while it is true that the physician and the dentist must frequently play the part of a simple spectator of the saddest incidents of their profession, the widely increased resources of modern science enable them, more than ever before, to restore new harmony to nature when it has become, in the language of Shakspeare, "like sweet bells jangled and out of tune."

In entering upon your professional life your chief and natural impulse will be to excel. But do not carry to the operating chair any ideality. Be practical. Make no experiments. You will probably be confronted by new conditions, but let them be subservient to the instruction and experience you have acquired here. Obey the old conservative rules until your pulse is strong enough to beat with a sense of personal independence and a judgment of your own. Then endeavor to keep pace with the world's thinkers. Promulgate ideas if the genius or the opportunity moves, but be prepared to fortify them. Remember, however, always, "that the race is not to the swift nor the battle to the strong."

If you are brought in contact with the poor do not neglect them. In the Pickwick Papers Bob Sawyer is made to say: "It is wonderful how the poor people patronize me. They call me up at all hours of the night. They take medicine to an extent which I should have conceived impossible. They put on leeches and blisters with a perseverance worthy of a better cause. They make additions to their family in a manner that is quite awful—six of those little promissory notes all due on the same day and all entrusted to me. It's all gratifying, but not so much so as the confidence of patients with a shilling to spare would be. It's a practice, and a very extensive practice, but that's all."

You will without doubt have some such experience, for it is as true in real life as in the imaginary sketch. But when you are brought face to face with it, whether in early or later practice, remember that as a Christian, sympathetic man, your business is with the poor as much as with the rich; that you are an almoner of the Almighty, and in dispensing His good and gracious gif.s, whether it be one or another of the ten talents referred to in the sacred writings, that you represent the highest intellectual type of Christian manhood.

When the old doctor knelt by the side of the dying waif, and, clasping the little white hand in his own, taught him to say, "Our Father which art in Heaven," while he led him towards the dark gate that all of us must enter, he crystallized religion and all its faith. And was it not natural that his old wife should pay him this tribute: "I never walk out with my husband but I hear the people bless him. I never go into a house of any degree but I hear his praises or see them in grateful eyes. I never lie down at night but I know that in the course of that day he has alleviated pain and soothed some fellow-creatures in the time of need. I know that from the beds of those who were past recovery thanks have gone up in the last hour for his patient administration."

I could not resist an impulse to pay a tribute to that department which has contributed so largely to your success this day. In the discharge of your professional duties you also will find rewards far greater than any pecuniary compensation, and will realize that happiness will cling to a man's good deeds as his shadow follows him in the sunshine.

Let me again, therefore, urge you with all earnestness to be true to the traditions of your adopted profession. Be worthy of the men whose lives have afforded you such brilliant examples. Be mindful of the honor of the institution from which you go forth to-day, under auspices so inspiring to ambitious manhood. Be proud of the noble body of professors whose intellectual and social lives during your period of study have been intertwined with your own. Excel them if you can.

Finally, be assured that while you maintain principles such as it has been sought to inculcate here and promote the best influences of your calling, your State and country will always be proud to do you honor as a graduate of the dental department of the University of Maryland.

